# Mutation in the two-component regulator BaeSR mediates cefiderocol resistance and enhances virulence in *Acinetobacter baumannii*

**DOI:** 10.1128/msystems.01291-22

**Published:** 2023-06-22

**Authors:** Xiaochen Liu, Yunjie Chang, Qingye Xu, Wang Zhang, Zhen Huang, Linyue Zhang, Shanshan Weng, Sebastian Leptihn, Yan Jiang, Yunsong Yu, Xiaoting Hua

**Affiliations:** 1 Department of Infectious Diseases, Sir Run Run Shaw Hospital, Zhejiang University School of Medicine, Hangzhou, Zhejiang, China; 2 Key Laboratory of Microbial Technology and Bioinformatics of Zhejiang Province, Hangzhou, Zhejiang, China; 3 Regional Medical Center for National Institute of Respiratory Diseases, Sir Run Run Shaw Hospital, School of Medicine, Zhejiang University, Hangzhou, Zhejiang, China; 4 Center of Cryo Electron Microscopy, Zhejiang University, Hangzhou, Zhejiang, China; 5 Department of Biophysics, Zhejiang University School of Medicine, Hangzhou, Zhejiang, China; 6 Zhejiang Provincial People's Hospital, People's Hospital of Hangzhou Medical College, Hangzhou, Zhejiang, China; 7 College of Agriculture and Biotechnology, Zhejiang University, Hangzhou, Zhejiang, China; 8 Zhejiang University-University of Edinburgh Institute, Zhejiang University, Haining, Zhejiang, China; 9 University of Edinburgh Medical School, Biomedical Sciences, College of Medicine and Veterinary Medicine, The University of Edinburgh, Edinburgh, United Kingdom; Institute of Urban Environment, Chinese Academy of Sciences, Xiamen, China

**Keywords:** BaeSR, cefiderocol resistance, MFS, MacAB-TolC, Csu pili, virulence

## Abstract

*Acinetobacter baumannii* has become one of the most challenging pathogens in many countries with limited treatment options available. Cefiderocol, a novel siderophore-conjugated cephalosporin, shows potent *in vitro* activity against *A. baumannii*, including isolates resistant to carbapenems. To date, few reports on the mechanisms of cefiderocol resistance are available. In order to investigate potential mechanisms of cefiderocol resistance in *A. baumannii*, we performed *in vitro* evolution experiments at sub-lethal concentrations of the antibiotic. All four cefiderocol-resistant strains obtained harbored mutations in two-component system BaeS-BaeR. When we engineered the mutations of BaeS (D89V) and BaeR (S104N) into the genome of ATCC 17978, these mutations increased cefiderocol minimum inhibitory concentrations (MICs) by 8-fold to 16-fold. Transcriptome analyses showed that the expression of MacAB-TolC and MFS transporters was up-regulated in BaeSR mutants. Strains over-expressing MFS transporter and MacAB-TolC displayed higher MICs and higher median inhibition concentration (IC_50_) values, while MICs and IC_50_ decreased when efflux pump genes were knocked out. In a BaeR mutant with up-regulated *csu* operon, we observed a higher number of pili, enhanced surface motility, and increased biofilm formation compared to wild-type ATCC 17978. Using the *Galleria mellonella* infection model, we found that the BaeS mutant in which *paa* operon was up-regulated exhibited increased virulence. In conclusion, the mutations in BaeSR decreased cefiderocol susceptibility of *A. baumannii* through up-regulating efflux pumps gene expression. BaeS or BaeR also controls the expression of *csu* and *paa*, influencing biofilm formation, surface motility, and virulence in *A. baumannii*.

**IMPORTANCE:**

The widespread prevalence of multi-drug-resistant *A. baumannii* (MDRAB) poses a significant therapeutic challenge. Cefiderocol is considered a promising antibiotic for the treatment of MDRAB infections. Therefore, it is necessary to study the potential resistance mechanisms of cefiderocol to delay the development of bacterial resistance. Here, we demonstrated that mutations in *baeS* and *baeR* reduced the susceptibility of *A. baumannii* to cefiderocol by up-regulating the expression of the MFS family efflux pump and MacAB-TolC efflux pump. We propose that BaeS mutants increase bacterial virulence by up-regulating the expression of the *paa* operon. This also reports the regulatory effect of BaeSR on *csu* operon for the first time. This study provides further insights into the role of BaeSR in developing cefiderocol resistance and virulence in *A. baumannii*.

## INTRODUCTION

*Acinetobacter baumannii* is an opportunistic nosocomial pathogen, which causes wound infections, pneumonia, and bacteremia, and is associated with high mortality. *A. baumannii* strains with extensive antibiotic resistance continue to emerge, which led to the bacterium being listed as a priority-1 pathogen, for which the development of efficient antibiotics is urgent (1). *A. baumannii* was the fifth most common bacterial species isolated from hospitals in China with over 65% strains resistant to carbapenem in 2021, according to the data of China antimicrobial surveillance network (CHINET). With such a critical situation, more treatment options are needed to address the current antimicrobial resistance crisis.

Cefiderocol is a novel siderophore-conjugated cephalosporin which enters bacteria by an active uptake mechanism via the iron transport system (2). The compound has excellent *in vitro* activity against a variety of Gram-negative bacteria, including *A. baumannii* (3, 4). Thus, cefiderocol is considered one of the therapeutic strategies for *A. baumannii* infections and was approved by the Food and Drug Administration (FDA) in 2019 to treat hospital-acquired pneumonia and ventilator-associated pneumonia caused by *A. baumannii* complex.

For most *A. baumannii* strains, resistance to cefiderocol is mediated by the production of β-lactamases, including NDM (NDM-1, NDM-5) and PER (PER-1, PER-7) (5). Another way to increase strains’ resistance is via the reduced expression or mutations of the siderophore receptor genes *pirA* or *piuA*, mediating the uptake of cefiderocol (6, 7). Possibly linked to cefiderocol minimum inhibitory concentration (MIC) increases in *Klebsiella pneumoniae* are mutations in the two-component regulation system BaeSR, which was reported in a non-clinical study (8). However, possible mechanisms associated with BaeSR mutations for the reduced susceptibility to cefiderocol have not been elucidated.

Bacterial two-component systems (TCSs) usually consist of a sensor and a response regulator, which play an important role in the regulation of signal transduction and the adaptation to environmental stimuli (9). At least 18 TCSs have been detected in the *A. baumannii* genome thus far (10). Among them, AdeRS, BaeSR, BfmRS, GacSA, and PmrAB have been well-characterized and associated with antibiotic resistance, virulence, biofilm formation, and aromatic compound catabolism (9). BaeS is a sensor embedded in the inner membrane of bacteria with histidine kinase activity. It detects physical and chemical changes in the external environment and transfers phosphate molecules to BaeR. BaeR is the cytoplasmic regulator, which then modulates the downstream gene expression (11). TCS BaeSR widely distributes in *Moraxellaceae*, *Enterobacterales*, *Pseuomonadales*, and *Burholderiales*, including *A. baumannii*, *Escherichia coli*, *Salmonella enterica* serovar Typhimurium (10, 12). A genome-wide analysis of the *E. coli* gene expression showed that BaeSR, functioning as a regulator of global transcription, regulates the expression of genes involved in multidrug transport, flagellum biosynthesis, chemotaxis, and maltose transport (13). Previous studies demonstrated that BaeSR influences tigecycline susceptibility in *A. baumannii* through regulating *adeAB* genes, which are generally considered to be regulated by AdeRS (14). The overlapping regulons of AdeRS and BaeSR could also mean that BaeSR functions through cross-talk with AdeRS (9). However, the relationship between BaeSR and cefiderocol resistance has not been explored so far.

In this study, we obtained cefiderocol-resistant *A. baumannii* strains through *in vitro* evolution experiments at sub-lethal concentrations of the antibiotic and investigated potential mechanisms of cefiderocol resistance in *A. baumannii*.

## RESULTS

### Mutations detected in cefiderocol-induced-resistant *A. baumannii* strains

To explore the potential resistance mechanisms in *A. baumannii* to cefiderocol, we performed *in vitro* evolution experiments in which we exposed the reference strain *A. baumannii* ATCC 17978 to sub-lethal concentrations of cefiderocol. After increasing the cefiderocol concentration over several passages, we obtained four cefiderocol-resistant *A. baumannii* strains, denoted as XH1799, XH1800, XH1823, and XH1824. These strains were whole genome sequenced. DNA-Seq reads of every strain were assembled to less than 100 contigs, with more than 99% coverage (Table S1). Then the mutations were identified in reference to the parental strain ATCC 17978. Mutations in BaeS (G212D, A71T, D89V, D360N) or BaeR (S104N) appeared in all four cefiderocol-induced-resistant strains (Table 1).

**TABLE 1:** Mutations in the cefiderocol-induced resistant strains

Strains	Position	Mutation^*a*^	Annotation	Gene	Description
XH1823	748418	A**G**T→A**A**T	S104N	AUO97_RS03395 ←	BaeR
XH1799	749772	G**G**T→G**A**T	G212D	AUO97_RS03400 ←	BaeS
	750196	**G**CG→**A**CG	A71T	AUO97_RS03400 ←	BaeS
XH1824	750141	G**A**C→G**T**C	D89V	AUO97_RS03400 ←	BaeS
XH1800	749329	**G**AT→**A**AT	D360N	AUO97_RS03400 ←	BaeS

^*a*^ Bold characters represent mutated nucleotide.

We constructed the mutations BaeS (D89V) and BaeR (S104N) in the genome of the parental strain ATCC 17978 by employing genetic engineering. The obtained mutants allowed us to investigate the impact of single mutations on cefiderocol susceptibility. Antimicrobial susceptibility testing showed a 16-fold cefiderocol MIC increase in the strains ATCC 17978 BaeR^S104N^. The BaeS (D89V) mutation increased the MIC by eightfold, from 0.125 to 1 μg/mL.

To clarify the influence of mutations on BaeS or BaeR function, we knocked out gene *baeS*, *baeR*, or both *baeS* and *baeR* of wild-type ATCC 17978. Meanwhile, we introduced pYMAb2-BaeS^D89V^ or pYMAb2-BaeR^S104N^ into wild-type ATCC 17978, and introduced pYMAb2-BaeS^WT^ or pYMAb2-BaeR^WT^ into the mutants, respectively. Cefiderocol MICs reduced from 0.125 to 0.03 μg/mL for BaeSR knockout strains. However, cefiderocol MICs increased when mutated BaeS or BaeR were expressed in wild-type ATCC 17978. Cefiderocol MICs had no obvious changes when we expressed wild-type BaeS or BaeR in BaeSR mutants (Table S2). These results indicated that mutations in BaeS or BaeR were gain-of-function.

In order to explore the effect of mutations on the susceptibility of other antibiotics, we tested the MICs of ceftazidime, cefepime, imipenem, meropenem, tigecycline, polymyxin, amikacin, ciprofloxacin, and erythromycin against cefiderocol-induced-resistant strains and BaeSR mutants. Compared with wild-type ATCC 17978, almost all antibiotics tested showed reduced susceptibility to induced-resistant strains XH1823 and XH1824. However, except tigecycline, there was no obvious susceptibility alterations for BaeSR mutants and BaeSR knockout strains. BaeS knockout caused a slight decrease in tigecycline MIC (Table S3). There may be unidentified genetic or epigenetic determinants of resistance in cefiderocol-induced-resistant strains XH1823 and XH1824, in addition to BaeSR mutation. These unidentified determinants lead to additional levels of resistance to cefiderocol and other antimicrobials, such as β-lactams and aminoglycosides.

### Transcriptome analysis of BaeS and BaeR mutants

**FIG 1:**
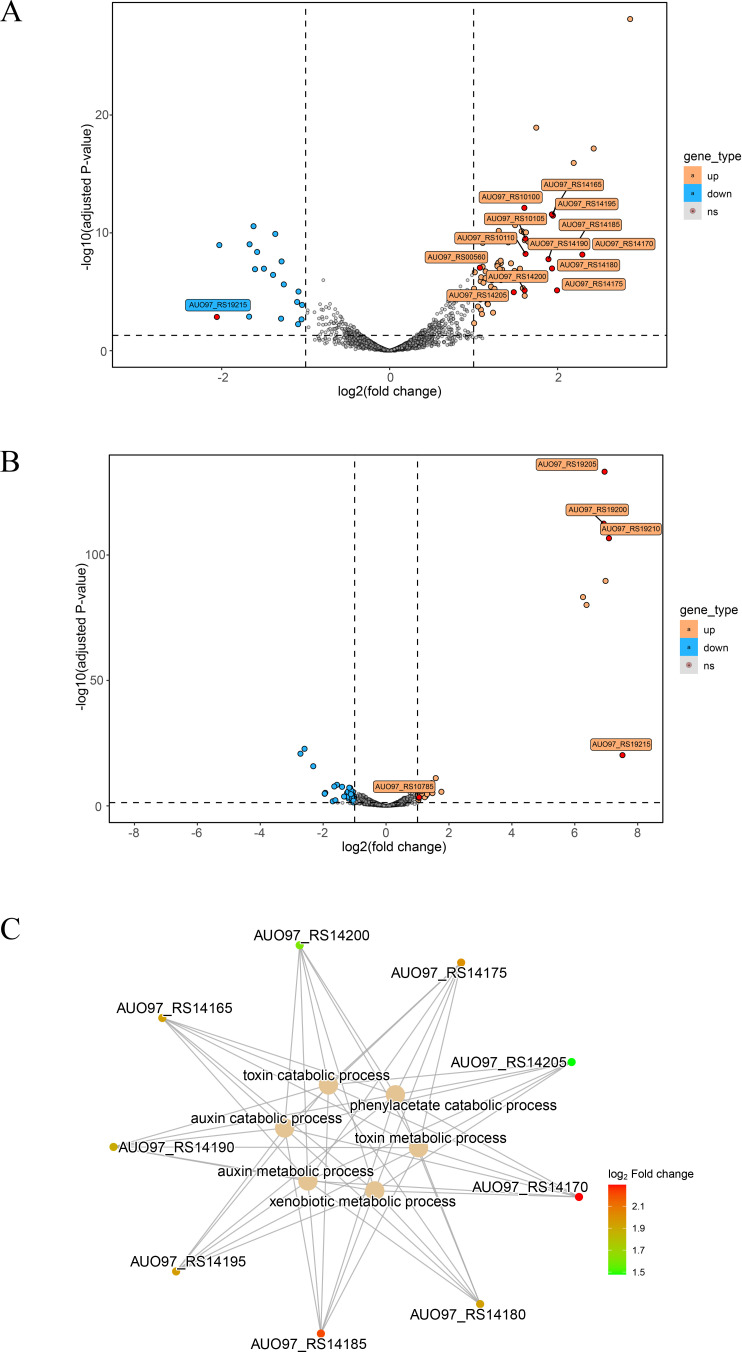
Transcriptional changes in ATCC 17978 BaeS^D89V^ and ATCC 17978 BaeR^S104N^. The volcano plot of differentially expressed genes identified by RNA-Seq in ATCC 17978 BaeS^D89V^ (**A**) and ATCC 17978 BaeR^S104N^ (**B**) compared to wild-type ATCC 17978. The orange dots represent the up-regulated genes with statistical significance (*P* < 0.05 and |log2(FC)| ≥1). The blue dots represent the down-regulated genes, while the gray ones represent genes with no statistical significance. Efflux pump coding genes, the *csu* operon, and the *paa* operon were marked in red. (**C**) The corresponding relationship between genes and significantly enriched GO terms in BaeS^S104N^ mutant.

In order to clarify the mechanism of the decrease in cefiderocol susceptibility caused by mutations in BaeSR, we compared the transcriptome of ATCC 17978 BaeS^D89V^ and ATCC 17978 BaeR^S104N^ with the wild-type ATCC 17978. The RNA-seq reads were aligned with the genome sequence of *A. baumannii* wild-type ATCC 17978 (GenBank accession no. NZ_CP018664.1). The analysis was based on a fold-change > 2 or < −2 (*P* < 0.05), where we observed a total of 77 differentially expressed genes in ATCC 17978 BaeS^D89V^, including 62 up-regulated genes and 15 down-regulated genes (Fig. 1A). In the case of ATCC 17978 BaeR^S104N^, we found 72 differentially expressed genes, of which 41 were up-regulated, and 31 were down-regulated (Fig. 1B). The Gene Ontology (GO) enrichment analysis of differentially expressed genes showed no significantly enriched GO terms with *P* < 0.05 in the BaeR mutant (Fig. S1B). In contrast, the top 15 significantly enriched terms in the BaeS mutant are shown in Fig. S1A, including toxin metabolic and catabolic processes. As shown in Fig. 1C and Table 2, genes in *paa* operon were up-regulated in the BaeS mutant, which encode enzymes involved in the phenylacetate pathway and are associated with toxin metabolic processes.

**TABLE 2:** Differentially expressed genes in ATCC17978 BaeS ^D89V^ and ATCC17978 BaeR ^S104N^ compared with ATCC 17978

Locus	Products	log_2_ fold-change	*P*-value
**ATCC17978 BaeS** ^**D89V**^			
AUO97_RS00560	MFS transporter	1.074	<0.0001
AUO97_RS10100	AdeC/AdeK/OprM family multidrug efflux complex outer membrane factor	1.604	<0.0001
AUO97_RS10105	MacB family efflux pump subunit	1.617	<0.0001
AUO97_RS10110	MacA family efflux pump subunit	1.618	<0.0001
AUO97_RS14165	Phenylacetic acid degradation bifunctional protein PaaZ	1.932	<0.0001
AUO97_RS14170	1,2-phenylacetyl-CoA epoxidase subunit A, PaaA	2.295	<0.0001
AUO97_RS14175	1,2-phenylacetyl-CoA epoxidase subunit B, PaaB	1.993	<0.0001
AUO97_RS14180	Phenylacetate-CoA oxygenase subunit PaaI	1.934	<0.0001
AUO97_RS14185	Phenylacetate-CoA oxygenase subunit PaaJ	2.213	<0.0001
AUO97_RS14190	Phenylacetate-CoA oxygenase/reductase subunit PaaK	1.891	<0.0001
AUO97_RS14195	Enoyl-CoA hydratase	1.944	<0.0001
AUO97_RS14200	2-(1,2-epoxy-1,2-dihydrophenyl) acetyl-CoA isomerase	1.607	<0.0001
AUO97_RS14205	3-hydroxyacyl-CoA dehydrogenase	1.478	<0.0001
AUO97_RS19215	CsuA/B	−2.057	0.0014
**ATCC17978 BaeR** ^**S104N**^			
AUO97_RS10785	MFS transporter	1.049	<0.0001
AUO97_RS19200	Molecular chaperone CsuC	6.919	<0.0001
AUO97_RS19205	CsuB	6.948	<0.0001
AUO97_RS19210	CsuA	7.086	<0.0001
AUO97_RS19215	CsuA/B	7.517	<0.0001

Among these differentially expressed genes, AUO97_RS10110, AUO97_RS10105, and AUO97_RS10100 coding for efflux pumps MacAB-TolC, and a major facilitator superfamily (MFS) efflux pump coding gene (AUO97_RS00560) were up-regulated in ATCC 17978 BaeS^D89V^. Another MFS efflux pump locus on AUO97_RS10785 in ATCC 17978 BaeR^S104N^ was 2.07-fold up-regulated compared to ATCC 17978. To our surprise, genes coding pilus protein expressed differentially in both ATCC 17978 BaeS^D89V^ and ATCC 17978 BaeR^S104N^. *csuA/B* was down-regulated in ATCC 17978 BaeS^D89V^, while *csuA/B*, *csuA*, *csuB,* and *csuC* were up-regulated in ATCC 17978 BaeR^S104N^ (Table 2).

### Up-regulation of efflux pumps genes is associated with increased cefiderocol MICs

**FIG 2:**
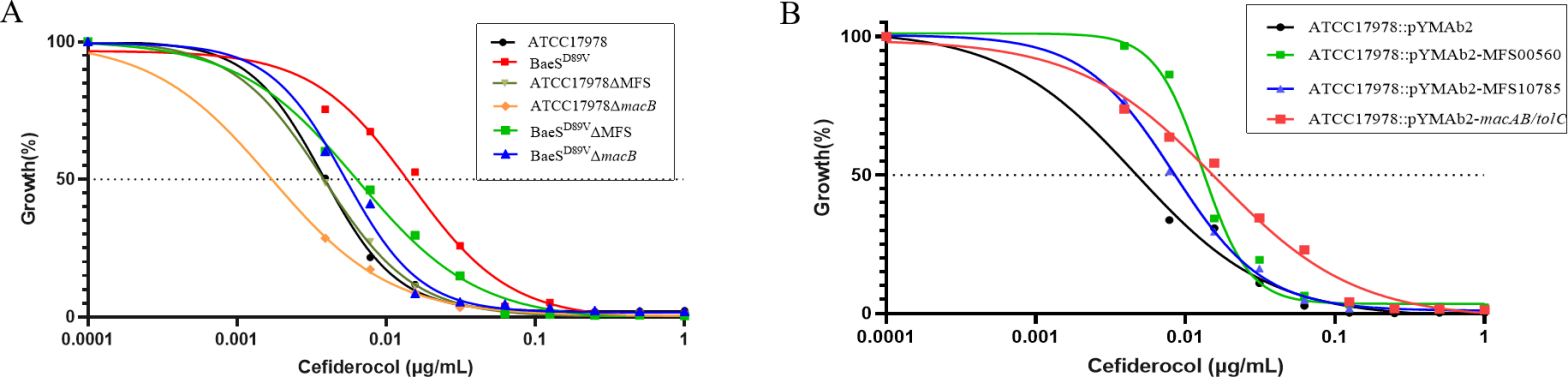
Cefiderocol dose–response curves. lg (cefiderocol concentration) is represented on the x-axis, and OD_600_ values at different concentrations of cefiderocol/OD_600_ values without cefiderocol ×100% are shown on the y-axis for nonlinear regression. The data points are the average values of three independent repeated experiments. (**A**) IC_50_ of efflux pumps knockout strains. (**B**) IC_50_ of efflux pumps over-expression strains.

As we observed high expression levels of efflux pump related genes, we determined the impact of these genes on cefiderocol susceptibility. Thus, we carried out gene knockout and over-expression experiments based on ATCC 17978. We confirmed the over-expression of *macAB-tolC*, AUO97_RS00560, and AUO97_RS10785 through RT-qPCR (Fig. S2). The BaeS mutant, in which we knocked out either *macB* or gene AUO97_RS00560, or both *macB* and gene AUO97_RS00560 showed slight but distinguishable decreases in cefiderocol MICs. However, cefiderocol MIC did not significantly decrease when we knocked out these genes from the genome of wild-type ATCC 17978, which indicated that the up-regulation of the efflux pump genes caused by the mutation in BaeS resulted in the reduction in cefiderocol susceptibility (Table S2). The cefiderocol MICs of ATCC 17978 strains with over-expression efflux pumps for pYMAb2-p*_ompA_-macAB-tolC,* pYMAb2-p*_ompA_*-MFS00560, or pYMAb2-p*_ompA_-macAB/tolC*-MFS00560 increased onefold to twofold (Table 3; Table S2). Considering that cefiderocol MIC may not be sensitive enough to detect slight changes in antibiotic susceptibility, we measured the IC_50_ values for cefiderocol in these strains. Here, we observed obvious changes, especially in the *macB* knockout strain, which showed increased susceptibility to cefiderocol, while the *macAB-tolC* over-expressing strain exhibited dramatically increased resistance (Table 3 and Fig. 2).

**TABLE 3:** MIC and IC_50_ of cefiderocol against induced-resistant strains and recombinant strains^*a*^

Strain	MIC			IC_50_
	FDC (μg/mL）	FDC/CCCP (μg/mL）	CCCP (μM)	(μg/mL）
ATCC17978	0.125	0.06	200	0.004
XH1823	>16	1	100	-
XH1824	>16	2	50	-
ATCC17978 BaeS^D89V^	1	0.25	200	0.015
ATCC17978 BaeR^S104N^	2	0.25	200	0.027
ATCC17978 ΔMFS00560	0.125	0.06	200	0.004
ATCC17978 Δ*macB*	0.06	0.03	200	0.002
ATCC17978BaeS^D89V^ ΔMFS00560	0.5	0.125	100	0.006
ATCC17978BaeS^D89V^ Δ*macB*	0.5	0.06	200	0.005
ATCC17978::pYMAb2	0.125	0.06	200	0.005
ATCC17978::pYMAb2-MFS00560	0.25	0.06	200	0.013
ATCC17978::pYMAb2-MFS10785	0.125	0.06	200	0.008
ATCC17978::pYMAb2-*macAB-tolC*	0.5	0.25	200	0.016

^*a*^ The concentration of CCCP was 25 μΜ. FDC, cefiderocol; CCCP, carbonyl cyanide m-chlorophenylhydrazine.

### The efflux pump inhibitor CCCP restores the susceptibility of *A. baumannii* to cefiderocol

To demonstrate that it is indeed the action of the efflux pumps that confer cefiderocol resistance, we used carbonyl cyanide m-chlorophenylhydrazine (CCCP) as an inhibitor. In the presence of 25 μM CCCP, MICs to cefiderocol were significantly reduced from ≥16 to 1 µg/mL for the strains XH1823 and XH1824. The MIC values of ATCC 17978 BaeS^D89V^ and ATCC 17978 BaeR^S104N^ were reduced to 0.25 μg/mL with a fourfold and eightfold reduction when CCCP was added (Table 3). Moreover, cefiderocol MICs of strains in which MFS efflux pumps were over-expressed also decreased when CCCP was added. Therefore, up-regulation of efflux pumps, driven by the proton motive force, appears to be a key mechanism for reduced susceptibility to the antibiotic in *A. baumannii* with BaeSR mutations. However, the MIC of strains with MacAB-TolC over-expression showed no obvious change in the presence of CCCP, which might be explained by the fact that CCCP could not inhibit the activity of MacAB-TolC.

### *A. baumannii* ATCC 17978 with a D89V mutation in BaeS exhibits increased virulence

**FIG 3:**
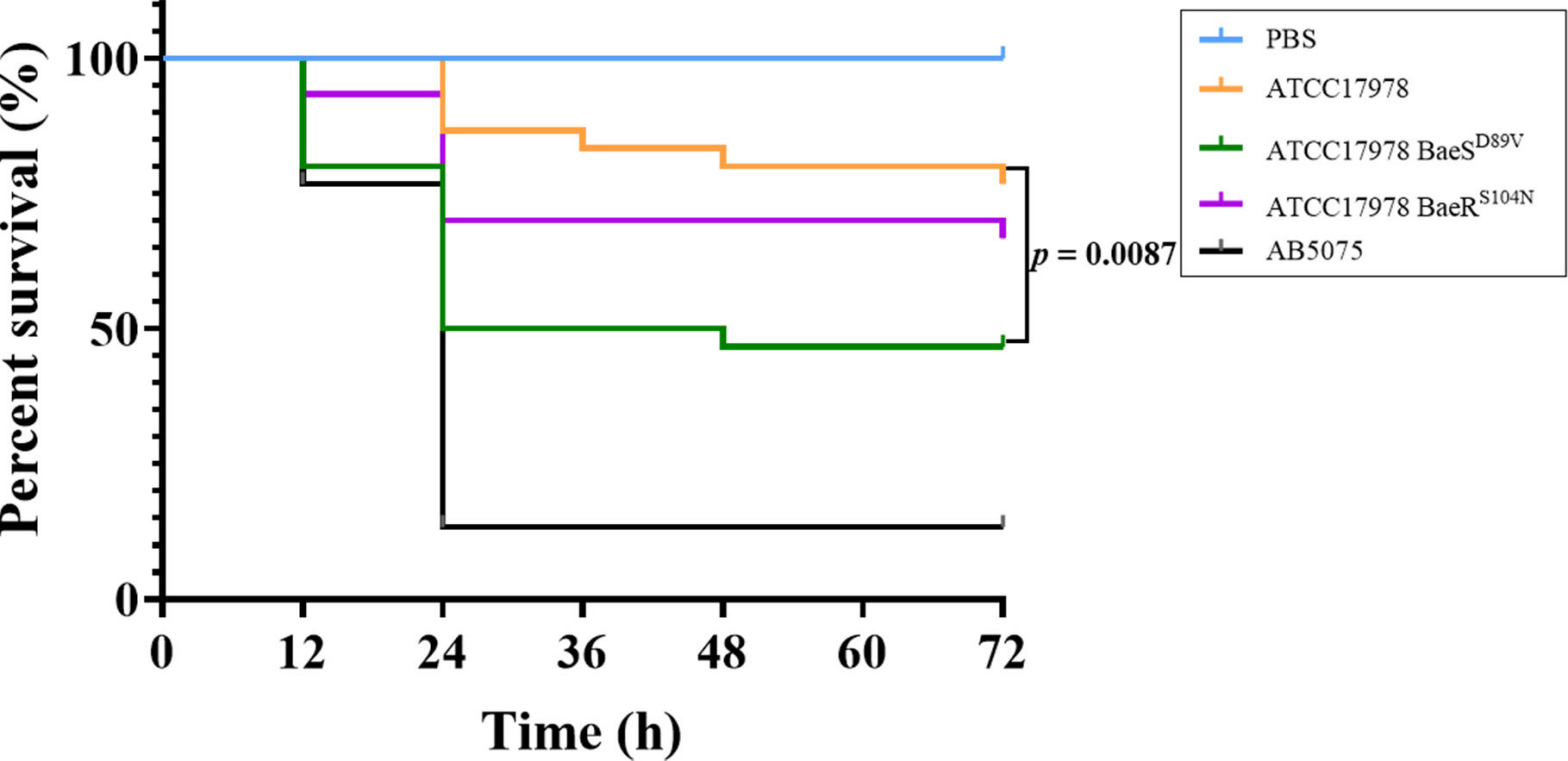
Kaplan–Meier survival curve analysis of bacterial virulence in *G. mellonella*. Phosphate buffered saline (PBS) is the negative control group; AB 5075 is the positive control group. The differences between groups were analyzed by a log-rank test. ATCC 17978 BaeS^D89V^ showed higher virulence than wild-type ATCC 17978 (*P* = 0.0087).

We previously found that the strain ATCC 17978 BaeS^D89V^ up-regulated the gene expression involved in toxin catabolic or metabolic processes. Thus, we used the *Galleria mellonella* infection model to evaluate the effect of BaeSR mutations on the virulence of *A. baumannii*. As shown in Fig. 3, ATCC 17978 BaeR^S104N^ showed similar virulence as ATCC 17978 (*P* = 0.3379). In contrast, the strain with the BaeS (D89V) mutation appeared to be significantly more virulent, as less than half of the larvae survived 72 h post-infection (*P* = 0.0087). However, all tested strains exhibited less virulence than the highly virulent AB5075 strain we used as a control.

### Mutations in BaeS and BaeR impact motility and biofilm formation via the expression of *csu* genes

**FIG 4:**
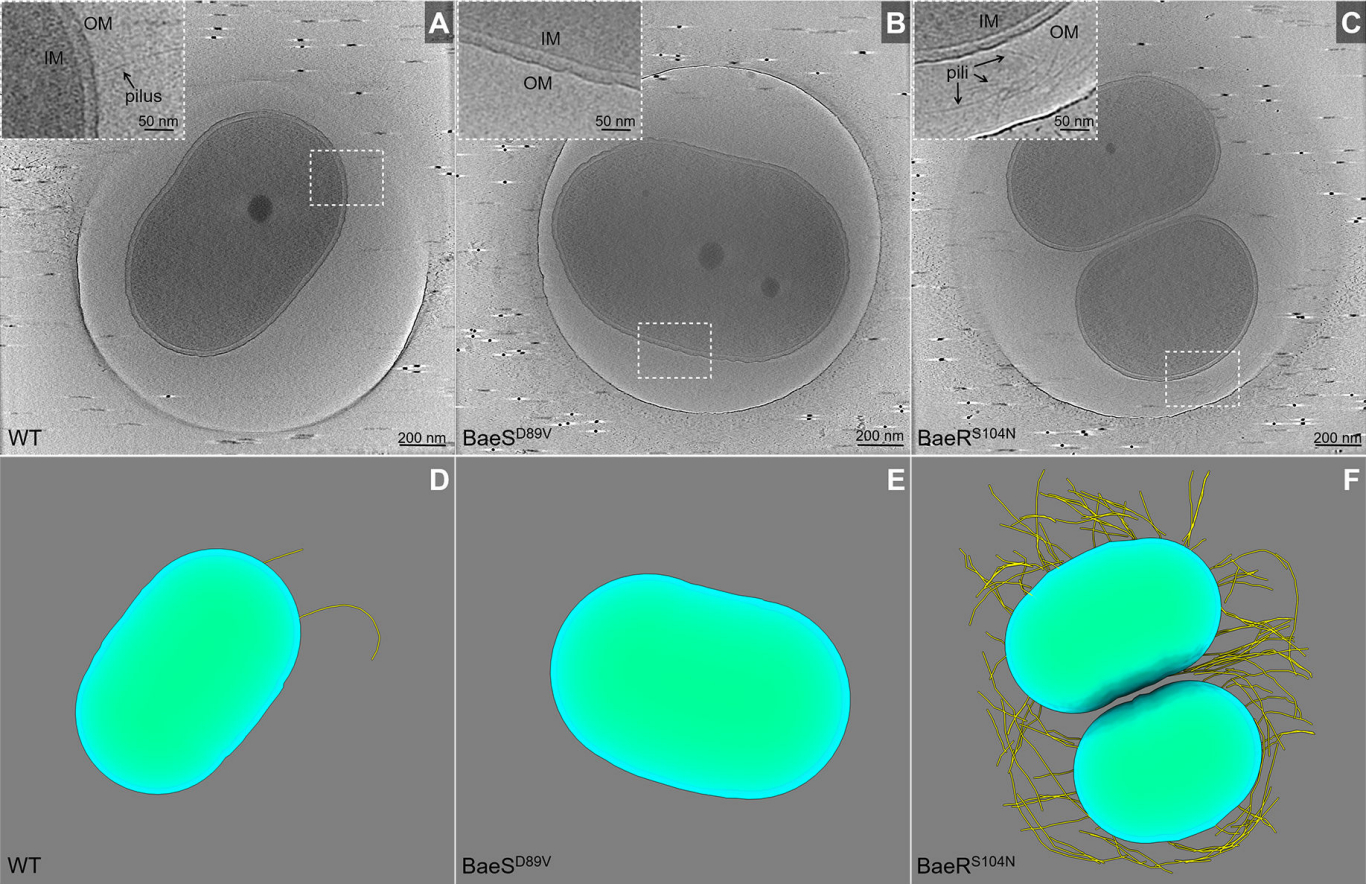
Representative cryo-ET images of ATCC 17978, ATCC 17978 BaeS^D89V^, and ATCC 17978 BaeR^S104N^. (**A–C**) Representative tomographic sections of wild-type ATCC 17978, BaeS mutant, and BaeR mutant, respectively. Insertions in (**A–C**) are the zoomed-in section for the dash-framed regions. (**D–F**) Corresponding sections with pili shown in yellow, the inner membrane (IM) shown in green, and the outer membrane (OM) shown in cyan. Pili were rarely observed in the reference strain ATCC 17978 (**A**) and the BaeS mutant (**B**). A much higher quantity was found in the BaeR mutant (**C**).

**FIG 5:**
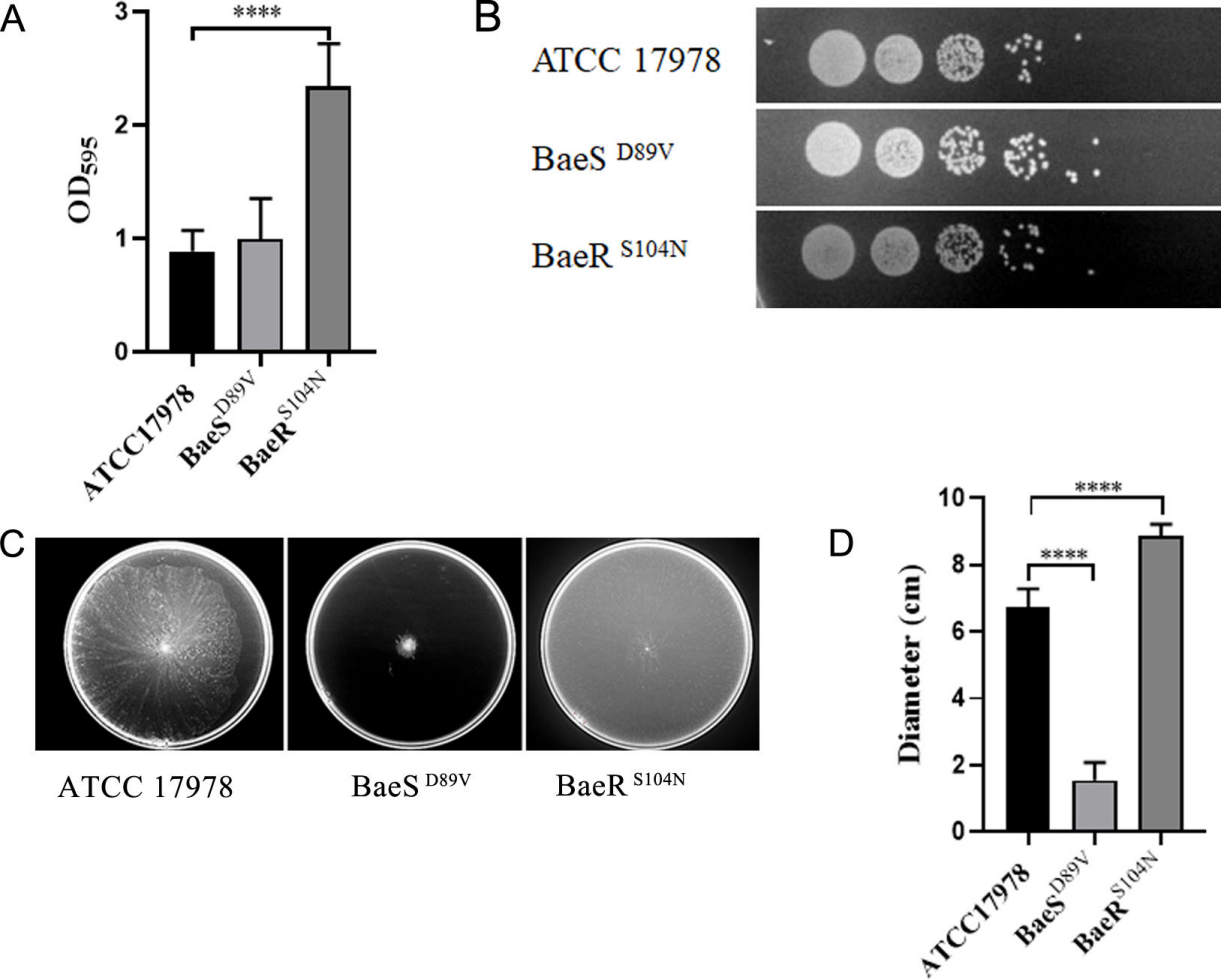
Biofilm formation and swarming motility changes of BaeSR mutants compared to the reference strain ATCC 17978. (**A**) Biofilm formation of *A. baumannii* ATCC 17978, ATCC 17978 BaeS^D89V^, ATCC 17978 BaeR^S104N^. (**B**) Attachment to HBE cells of *A. baumannii* ATCC 17978 and its BaeSR mutants. (**C**) Swarming motility of *A. baumannii* ATCC 17978 and its BaeSR mutants. And the diameters of growth were calculated in (**D**). ****P < 0.0001.

Genes coding for the pilus protein expressed differently in the BaeS mutant and the BaeR mutant. Using cryo-electron tomography (cryo-ET) analysis, we could observe that pili were rarely present in the case of the ATCC 17978 strain. Similarly, few pili were seen on the surface of the BaeS mutant. In contrast to these two strains, pili were abundant in the BaeR mutant (Fig. 4; Videos 1 to 3). When assessing the biofilm formation of mutants, we observed that ATCC 17978 BaeR^S104N^ had a greater ability to form biofilms on plastic surfaces than the parent strain (Fig. 5A), while ATCC 17978 BaeS^D89V^ did not show significant differences in comparison with the parent strain. We also tested bacterial adhesion to human bronchial epithelial (HBE) cells. No significant differences were detected between the BaeSR mutants and ATCC 17978 (Fig. S3; Fig. 5B). Next, we investigated the swarming motility of the strains as we observed an impact on the expression of *csu* genes, with ATCC 17978 BaeR^S104N^ leading to higher and ATCC 17978 BaeS^D89V^ to lower expression levels. The assays showed that the motility was enhanced for the strains with over-expressed Csu pili and almost halted in the BaeS^D89V^ mutant (Fig. 5C and D).

## DISCUSSION

As a global regulator, the main function of the TCS BaeSR is to up-regulate efflux pump expression in response to specific stress. BaeSR has been reported in *Salmonella Typhimurium* and *E. coli* to modulate the expression of outer membrane proteins or specific efflux pumps, thus affecting antibiotic susceptibility (15 - 17). In *A. baumannii,* BaeSR has been associated with tigecycline resistance via regulating AdeIJK and AdeABC pumps (14, 18). In this study, ATCC 17978 showed higher cefiderocol MICs when valine substituted aspartic acid at 89 of BaeS or asparagine substituted serine at 104 of BaeR. The 104th amino acid is located in the cheY-homologous receiver domain of BaeR, and the amino acid substitution at this position may affect the reception of upstream signals.

Transcriptome analysis showed that the BaeS mutation caused the over-expression of *macAB-tolC* and an MFS efflux pump coding gene AUO97_RS00560. Another MFS efflux pump, AUO97_RS10785, was up-regulated in strains with the BaeR mutation. Although previous reports describe the role of BaeSR as regulating the expression of resistance-nodulation-division (RND) efflux family (AdeABC and AdeIJK) or the ATP-binding cassette (ABC) family (MacAB-TolC), we demonstrated for the first time that the BaeSR participated in the regulation of the MFS transporter expression. MFS is by far the largest and most diverse of secondary transporters superfamily known (19), and its members are ubiquitous across all domains of life (20). MFS transporters in the important pathogen *A. baumannii* have not been studied in detail, with CraA, AmvA, AbaF, AbaQ, and TetA being associated with susceptibility of chloramphenicol, erythromycin, fosfomycin, quinolones, and tetracyclines, respectively (20 - 23).

In this study, we found that efflux pump inhibitor CCCP restored the susceptibility of BaeSR mutants to cefiderocol. The main mechanism of CCCP’s action was via disrupting the proton motive force of membranes. Thus, CCCP inhibited efflux pumps such as RND and MFS family, which were driven by the proton motive force (21). The compound inhibits the activity of MFS transporters, but not that of MacAB-TolC, which belongs to the ATP-driven ABC transporters. We showed that cefiderocol MICs of BaeSR mutants decreased significantly when CCCP was added, which provided clear evidence that MFS transporters played a role in the cefiderocol removal as a consequence of BaeSR mutations. The addition of CCCP decreased the cefiderocol MIC of *macB* knockout strain compared to ATCC17978 BaeS^D89V^, which indicated that MacAB-TolC and the MFS transporter potentially act together to allow bacterial survival in the presence of cefiderocol.

Although the transcriptome analysis and the efflux pumps inhibition assay confirmed that the up-regulation of certain efflux pumps played an important role in cefiderocol resistance, we did not observe the same effect when performing single-gene knockout and over-expression experiments. When we knocked out AUO97_RS00560 or *macB*, we only observed a slight reduction in MIC values. A possible reason is that other efflux pumps may be up-regulated when one efflux system is absent or inactive. Bacteria express numerous efflux transporters with functional redundancy and partially overlapping substrates. The expression of other alternative transporters may increased when one of them are disrupted (24). Another possibility should be mentioned: the fact that MFS transporters are energized inner membrane transporters, the simple over-expression might not be sufficient to remove cefiderocol from the periplasm to the surrounding media across the outer membrane, which might require a carefully balanced over-expression network containing MFS transporters, the periplasmic adaptor proteins and outer membrane factor channel-like proteins (25). In addition, even when we over-expressed both MFS transporter and MacAB-TolC at the same time, the MIC of cefiderocol was not as high as that of the BaeS mutant, which suggested that maybe factors other than the two pumps we did not find out also contributed to the decreased cefiderocol susceptibility.

**FIG 6:**
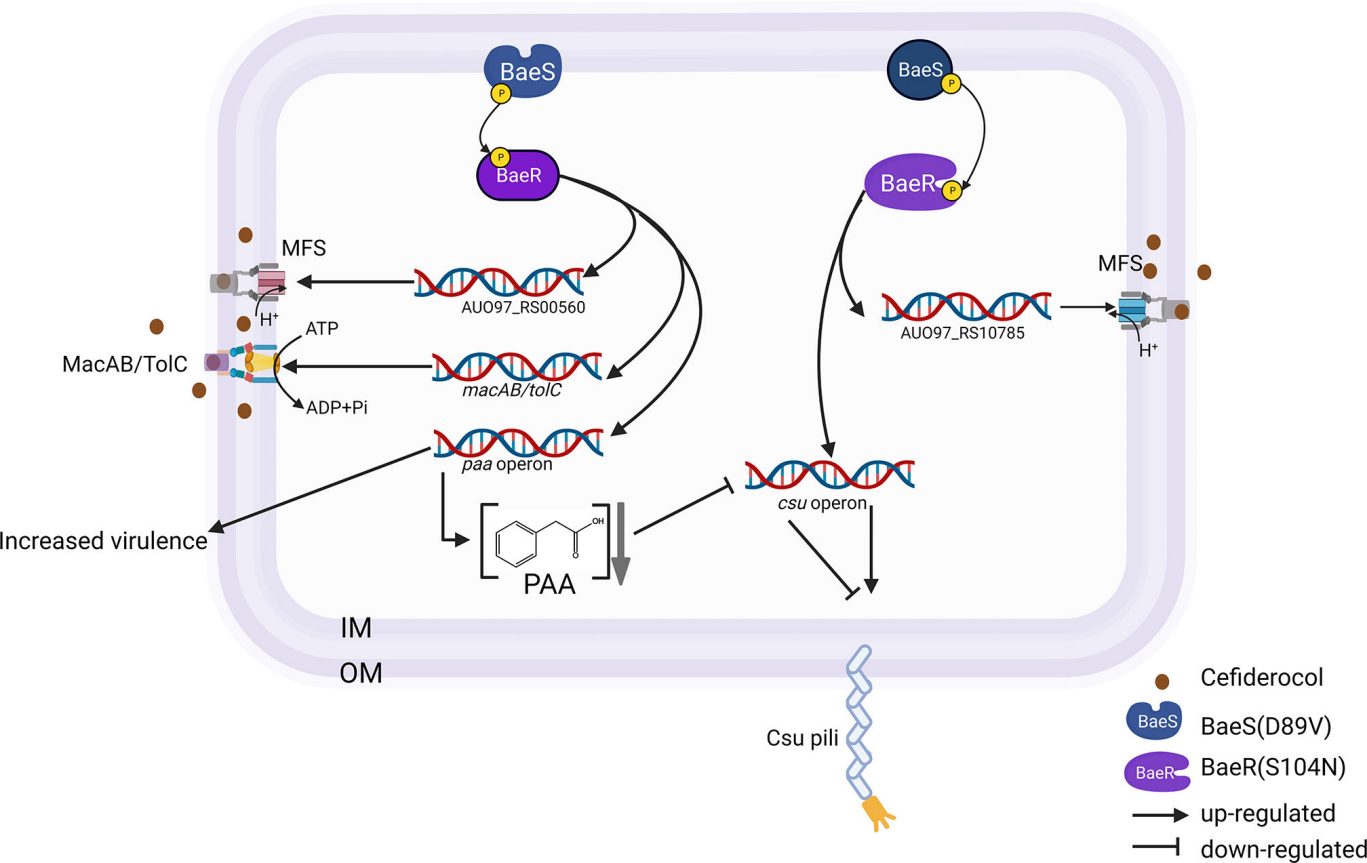
Proposed model of BaeSR regulation in *A. baumannii*. Mutation in BaeS (D89V) increases the expression of efflux pump coding genes and the *paa* operon. An over-expression of the *paa* operon leads to decreased PAA levels, which down-regulates the expression of the *csu* operon. Mutation in BaeR (S104N) up-regulates the expression of efflux pump coding gene AUO97_RS10785 and also leads to an increase in the expression of the *csu* operon that leads to more pili. Created with BioRender.com.

We also found *paa* operon up-regulated in the BaeS mutant. *paa* operon encodes enzymes responsible for phenylacetic acid (PAA) degradation, which is linked to bacterial virulence; it was previously shown that knocking out gene *paaA* in ATCC 17978 results in attenuated virulence in a zebrafish infection model (26). A previous study showed that the virulence of ATCC 17978 was attenuated in Δ*paaB* mutant in a murine catheter-associated urinary tract infection model (27). Consistent with this, we observed an increase in *A. baumannii* ATCC 17978 virulence in the BaeS mutant with up-regulated *paa* in the *G. mellonella* infection model. The study mentioned above also indicated that Csu expression nearly halved when the *paa* operon was over-expressed, and could be restored by adding an exogenous PAA (27). Similarly, we observed *paa* up-regulation, accompanied by *csu* down-regulation in the BaeS mutant strain. Therefore, we speculate that this process may be regulated by BaeS (Fig. 6).

The *csu* cluster encodes Csu pili, which are formed from four different subunits, namely CsuA/B, CsuA, CsuB, and CsuE, and are assembled via a novel archaic chaperone-usher pathway (28). Csu pili are required for biofilm formation on abiotic surfaces (29), but are not necessary for bacterial adhesion to epithelial cells (30). In our study, ATCC 17978 BaeR^S104N^ showed higher expression of the *csu* operon than the reference strain. This resulted in increased biofilm formation on plastic surfaces and enhanced motility on agar plates. However, the biofilm formation of ATCC 17978 BaeS^D89V^ with decreased expression of *csuA/B* was not significantly different from that of the reference strain. This may be because CsuA, CsuB, and CsuE subunits are still assembled to some form of short pili or tip fibrillum and mediate biofilm formation on abiotic surfaces, as described in a previous study (31). No difference in epithelial cell attachment between the reference strain and the BaeSR mutants was observed. BfmR, the cytoplasmic response regulator of TCS BfmSR, is a transcriptional activator that regulates the expression of *csu* operon (32). Another study found that A1S_2811, a two-component regulator in ATCC 17978, might influence surface motility and biofilm formation by regulating the *csu* operon (33). This suggests that the expression of *csu* is under the control of multiple transcriptional regulators. To our knowledge, this is the first time that BaeSR has been reported to regulate *csu* expression, which required further investigation into whether this function of BaeSR occurs via a cross-talk with other regulators.

In conclusion, mutations in BaeS (D89V) and BaeR (S104N) decrease the susceptibility to cefiderocol in *A. baumannii* via the up-regulation of MFS transporters and the efflux pump MacAB-TolC. In addition, the BaeS mutation increases virulence by up-regulating the *paa* operon. The TCS BaeSR plays a key role in regulating the expression of Csu pili, which affects biofilm formation and swarming motility. This study provides further insights into the role of BaeSR in cefiderocol resistance and virulence in *A. baumannii*.

## MATERIALS AND METHODS

### Bacterial isolates, culture conditions, and antimicrobial susceptibility testing

All isolates were cultured in Mueller–Hinton broth (Oxoid, UK), or on Mueller–Hinton agar plates at 37°C overnight. Minimum inhibitory concentrations (MICs) of cefiderocol were determined by broth microdilution method in cation-adjusted Mueller–Hinton broth or iron-depleted cation-adjusted Mueller–Hinton broth (ID-CAMHB) according to CLSI guidelines (34).

Half maximal inhibitory concentration (IC_50_) of cefiderocol was detected on a 96-well cell culture plate through microdilution broth method. Cefiderocol was serial diluted with 100 µL ID-CAMHB to reach a final concentration of 0.0039 µg/mL (from 4 µg/mL. Then ~5 × 10^5^ CFU/mL bacterial cells were added to each well. Bacteria cells were inoculated in the medium containing no cefiderocol as control. OD_600_ values of each well were measured using a Multiskan Go microplate reader (Thermo Scientific, USA) after incubation for 18 h. Three independent experiments were performed for every isolate. The OD_600_ average values of three independent experiments at different cefiderocol concentrations were used for nonlinear regression [log(inhibitor) vs response-Variable slope (four parameters)], and calculation of IC_50_ values by GraphPad Prism 8.0.2.

We added 25 μΜ efflux pump inhibitor CCCP into ID-CAMHB to inhibit the activity of efflux pumps and measured the cefiderocol MICs to prove the role of efflux pumps in cefiderocol resistance.

### *In vitro* evolution experiments for the selection of cefiderocol-resistant strains

*A. baumannii* ATCC 17978 served as a parental culture to perform serial passage experiments as previously described (35). Briefly, a single clone of ATCC 17978 was inoculated in 2 mL MH broth with an initial cefiderocol concentration of 0.5-fold MIC (0.06 µg/mL) and transferred into 2 mL fresh media containing a double concentration of cefiderocol at a dilution of 1:100 every 24 h until no further bacterial growth was observed. All the cultures in serial passage experiments were stored at −80°C. One single colony was randomly selected from the final populations of four parallel, serial passage experiments and stored as XH1799, XH1800, XH1823, and XH1824 (Table 4).

### Whole genome sequencing and analysis

The genomic DNA of XH1799, XH1800, XH1823, and XH1824 were extracted using QIAamp DNA Mini Kit (Qiagen, USA) according to the manufacturer. Zhejiang Tianke (Hangzhou, China) was entrusted with the whole genome sequencing (WGS) via Illumina Hiseq. With reference to the original genome of ATCC 17978 (GenBank accession no. NZ_CP018664), mapping and putative mutations detection were carried out using breseq v0.33.0 (36). Putative mutations were confirmed by PCR and Sanger sequencing (primers are listed in Table S4).

### Reconstruction of mutations

Reconstruction experiments were performed as described previously (37, 38). Fragment of *baeS* (D89V), and *baeR* (S104N) was amplified from corresponding induced-resistant strains. Purified PCR products were inserted into the pMo130-Hyg^r^ vector digested with *Bam*HI and *Xba*I using 2× Hieff Clone Enzyme Premix (Yeason, China). The recombinant plasmid was elecro-transformed into ATCC 17978 and selected on plates containing 100 µg/mL hygromycin. Then, ATCC 17978 harboring inserted pMo130-Hyg^r^-target fragment construct was cultured overnight in Luria-Bertani broth containing 20% sucrose to select isolates of which target gene achieved allelic replacement by a second cross-over (Table 4). The reconstructed mutations were confirmed through PCR and Sanger sequencing.

### Transcriptome analysis

To further clarify the regulatory effect of BaeS and BaeR mutations on gene expression, RNA-sequencing of ATCC 17978, ATCC 17978 BaeS^D89V^, and ATCC 17978 BaeR^S104N^ was performed. Briefly, the overnight bacterial solution was diluted in 100 mL MH broth at a 1: 100 and grown to log phase. The culture was centrifuged at 5,000 rpm for 10 min. After grinding with liquid nitrogen, total RNA was extracted using TRIZOL Reagent (Invitrogen, USA). Zhejiang Tianke (Hangzhou, China) was entrusted with bacteria mRNA sequence library construction and sequencing. The RNA-seq reads were mapped to the reference genome of *A. baumannii* ATCC 17978 using Rockhopper v2.0.3 (39). The output data were analyzed by edgeR (40). Differentially expressed genes were selected with significance cutoffs of BH adjusted *P* < 0.05 and |fold-change| ≥2. GO annotation was performed by eggNOG (http:// eggNOG-mapper.embl.de), and enrichment analysis was performed using ClusterProfiler R package (41).

### Gene knockout and over-expression

Knockout and over-expression experiments were performed to further verify the role of the high expression of efflux pumps in cefiderocol resistance. Gene knockout was performed according to the previous study (37). Briefly, amplify up-stream and down-stream fragments of target genes and clone purified PCR products into pMo130-Hyg^r^ vector digested with *Bam*HI and *Xba*I using 2× Hieff Clone MultiS Enzyme Premix (Yeason, China). The recombinant plasmid was electro-transformed into wild-type ATCC 17978 and selected on plates containing 100 µg/mL hygromycin. Then, ATCC 17978 harboring inserted pMo130-Hyg^r^-target fragment (Up/Down) construct was cultured in Luria-Bertani broth containing 20% sucrose to induce the second cross-over and allelic replacement. Select clones which were white when sprayed with 0.45 M pyrocathechol and were susceptible to 100 µg/mL hygromycin and confirm that the target gene was deleted via PCR and Sanger sequencing (primers were listed in Table S4).

Target genes together with *ompA* promoter were cloned into pYMAb2-Hyg^r^ plasmid digested with *Bam*HI and *Sal*І using 2× Hieff Clone MultiS Enzyme Premix (Yeason, China), and then electro-transformed into ATCC 17978 to over-express target genes in host bacteria (Table 4). qRT-PCR was used to verify the over-expression of the target genes. The expression of the *baeS*, *baeR*, AUO97_RS00560, AUO97_RS10785, and *macB* were assessed using specific primers (Table S4), employing real-time PCR using TB Green Premix Ex Taq (Takara, Japan) with standard procedure for two-step PCR amplification according to the instruction in the LightCycler 480 System (Roche Diagnostics). Three independent experiments were performed for every isolate.

### Motility assay, biofilm formation on abiotic surface, and HBE adhesion

Swarming motility assays were performed as described previously (42). Firstly, Luria-Bertani media containing 0.35% agar was poured into 9 cm Petri dishes. And then, a colony was inoculated in the center of the above-described medium and incubated at 37°C. The diameter of growth was determined after 14 h of incubation.

The biofilm formation assay was performed according to the previous description with minor modifications (43). Briefly, one colony was inoculated in MH broth overnight at 37°C, and then transferred into a fresh broth at a dilution of 1:100 in a 96-well cell culture plate and incubated overnight. Each culture was added to at least three wells. Adherent cells were washed with phosphate buffered saline (PBS), stained with 0.1% crystal violet at 4°C for 30 min, and washed with PBS three times. Use ethanol and shake for 30 min at 200 rpm to release the dye. Absorbance at 595 nm was measured on a Multiskan Go microplate reader. Three independent experiments were performed.

HBE cell line (#CRL2741; ATCC) grew in 5% CO_2_ at 37°C in Dulbecco’s Modification of Eagle’s Medium (DMEM, Corning, USA) with 10% fetal bovine serum as well as 1% penicillin/streptomycin. Monolayers were washed once with PBS lightly, and then log-phase bacteria were added to cells at a MOI of 100:1 and incubated for 1 h at 37°C. Infected monolayers were washed three times with PBS and lysed with 0.2% Triton X-100. Ten microliters of gradient diluted lysates were dropped onto MH agar plates. Count the number of clones to assess the attachment of BaeS and BaeR mutants to HBE cells.

### Cryo-electron tomography and three-dimensional visualization

ATCC 17978 and BaeSR mutants were cultured in Luria-Bertani broth overnight and were centrifuged at 5000 rpm for 10 min. The obtained pellets were suspended in PBS, and the cell concentration was adjusted to ~OD_600_ = 0.8. 5 µL of the cell suspension was placed on freshly glow-discharged (for 40 s) holey carbon grids (Quantifoil Cu *R2*/1,200 mesh) after adding 15 nm gold marker solution. The grids were blotted with Whatman filter paper and then rapidly frozen in liquid ethane, using a homemade plunger apparatus. The grids were imaged using a 200-kV electron microscope (Talos F200C, Thermo Fisher Scientific) equipped with a field emission gun and a fast-imaging Ceta camera (Thermo Fisher Scientific). Serial EM was used to collect all tilt series (44). Images were acquired at 22,000× magnification with an effective pixel size of 4.81 Å at the specimen level. The defocus was set as close to −5 µM. A total dose of ~90 e^−^/Å^2^ (the dose was estimated based on the screen current) is distributed among 35 tilt images covering angles from −51° to 51° with a tilt step of 3°. All recorded images were then stacked and aligned by IMOD (45) and tomograms (binned four times; 19.24 Å pixel size) were reconstructed by simultaneous iterative reconstruction technique (SIRT) reconstruction using Tomo3D (46). Segmentations of representative tomographic reconstructions from ATCC 17978, ATCC 17978 BaeS^D89V^, and ATCC 17978 BaeR^S104N^ cells were manually constructed using IMOD (45).

**TABLE 4:** Description of plasmids and bacterial strains used in this study

Plasmid or strain	Description	Source
**Plasmids**		
pYMAb2-Hyg^r^	*E. coli–A. baumannii* shuttle plasmid with hygromycin resistance cassette	Reference 47
pYMAb2-p*_ompA_*-BaeS^WT^	pYMAb2-Hyg^r^ with inserted promoter of *ompA* and wild-type gene *baeS*	This study
pYMAb2-p*_ompA_*-BaeR^WT^	pYMAb2-Hyg^r^ with inserted promoter of *ompA* and wild-type gene *baeR*	This study
pYMAb2-p*_ompA_*-BaeS^D89V^	pYMAb2-Hyg^r^ with inserted promoter of *ompA* and mutated gene *baeS* (D89V)	This study
pYMAb2-p*_ompA_*-BaeR^S104N^	pYMAb2-Hyg^r^ with inserted promoter of *ompA* and mutated gene *baeR* (S104N)	This study
pYMAb2-p*_ompA_*-MFS00560	pYMAb2-Hyg^r^ with inserted promoter of *ompA* and gene AUO97_RS00560	This study
pYMAb2-p*_ompA_*-MFS10785	pYMAb2-Hyg^r^ with inserted promoter of *ompA* and gene AUO97_RS10785	This study
pYMAb2-p*_ompA_-macAB-tolC*	pYMAb2-Hyg^r^ with inserted promoter of *ompA*, gene *macA*, *macB,* and *tolC*	This study
pYMAb2-p*_ompA_-macAB*/*tolC*-MFS00560	pYMAb2-Hyg^r^ with inserted promoter of *ompA* and gene *macA*, *macB, tolC,* and AUO97_RS00560	This study
pMo130-Hyg^r^	Suicide plasmid with hygromycin resistance cassette	Reference 37
**Strains**		
ATCC17978	*A. baumannii* model strain	
XH1823	Cefiderocol-resistant strain with BaeR mutation obtained from *in vitro* evolution experiment of ATCC17978	This study
XH1824	Cefiderocol-resistant strain with BaeS mutation obtained from *in vitro* evolution experiment of ATCC17978	This study
ATCC17978 BaeS^D89V^	BaeS (D89V) mutation recombinant strain based on the genetic background of ATCC17978	This study
ATCC17978 BaeR^S104N^	BaeR (S104N) mutation recombinant strain based on the genetic background of ATCC17978	This study
ATCC17978ΔBaeS	*baeS* gene knockout strain of ATCC17978	This study
ATCC17978ΔBaeR	*baeR* gene knockout strain of ATCC17978	This study
ATCC17978ΔBaeSR	*baeS* and *baeR* gene knockout strain of ATCC17978	This study
ATCC 17978::pYMAb2-BaeS^D89V^	Introduce pYMAb2-p_ompA_-BaeS^D89V^ into ATCC17978	This study
ATCC 17978::pYMAb2-BaeR^S104N^	Introduce pYMAb2-p_ompA_-BaeR^S104N^ into ATCC17978	This study
ATCC17978 BaeS^D89V^::pYMAb2-BaeS^WT^	Introduce pYMAb2-p_ompA_-BaeS^WT^ into ATCC17978 BaeS^D89V^	This study
ATCC17978 BaeR^S104N^::pYMAb2-BaeR^WT^	Introduce pYMAb2-p_ompA_-BaeR^WT^ into ATCC17978 BaeR^S104N^	This study
ATCC17978 ΔMFS00560	AUO97_RS00560 gene knockout strain of ATCC17978	This study
ATCC17978 Δ*macB*	*macB* gene knockout strain of ATCC17978	This study
ATCC17978 ΔMFS00560 Δ*macB*	AUO97_RS00560 and *macB* knockout strain of ATCC17978	This study
ATCC17978BaeS^D89V^ΔMFS00560	AUO97_RS00560 gene knockout strain of ATCC17978 BaeS^D89V^	This study
ATCC17978BaeS^D89V^Δ*macB*	*macB* gene knockout strain of ATCC17978 BaeS^D89V^	This study
ATCC17978BaeS^D89V^ ΔMFS00560Δ*macB*	AUO97_RS00560 and *macB* knockout strain of ATCC17978 BaeS^D89V^	This study
ATCC17978::pYMAb2-MFS00560	AUO97_RS00560 over-expression strain, introduce pYMAb2-p*_ompA_*-MFS00560 into ATCC17978	This study
ATCC17978::pYMAb2-MFS10785	AUO97_RS10785 over-expression strain, introduce pYMAb2-p*_ompA_*-MFS10785 into ATCC17978	This study
ATCC17978::pYMAb2-*macAB-tolC*	*macAB-tolC* over-expression strain, introduce pYMAb2-p*_ompA_-macAB-tolC* into ATCC17978	This study
ATCC17978::pYMAb2-*macAB*/*tolC*-MFS00560	*macAB-tolC* and AUO97_RS00560 over-expression strain, introduce pYMAb2-p*_ompA_-macAB/tolC*-MFS00560 into ATCC17978	This study
ATCC17978::pYMAb2	Introduce pYMAb2-Hyg^r^ into ATCC17978, as a control	This study

### *G. mellonella* infection model

The virulence of BaeSR mutants was evaluated using the *G. mellonella* infection model. Log-phase bacterial cultures were resuspended in PBS to 10^7^ CFU/mL. Thirty larvae of *G. mellonella* in each group were inoculated with 10 µL of bacterial suspension and incubated at 37°C. The death of *G. mellonella* was defined as no movement. Viability was assessed every 12 h for a total of 72 h.

## Data Availability

Raw DNA-Seq data sets of *A. baumannii* strains XH1799, XH1800, XH1823, and XH1824 have been deposited in Sequence Read Archive (BioProject accession no. PRJNA866888). RNA-Seq data sets are available at Sequence Read Archive under BioProject no. PRJNA866314.

## SUPPLEMENTAL MATERIAL

Video 1 msystems.01291-22-s0001.mov Representative cryo-ET images of wild-type ATCC 17978.

Video 2 msystems.01291-22-s0002.mov Representative cryo-ET images of ATCC 17978 BaeS (D89V).

Video 3 msystems.01291-22-s0003.mov Representative cryo-ET images of ATCC 17978 BaeR (S104N).

Fig. S1 msystems.01291-22-s0004.docx GO enrichment analysis of differentially expressed genes.

Fig. S2 msystems.01291-22-s0005.docx Relative expression of AUO97_RS00560, AUO97_RS10785, and macB in over-expression strains.

Fig. S3 msystems.01291-22-s0006.docx Human bronchial epithelial (HBE) adhesion of ATCC 17978 and its BaeSR mutants.

Table S1 msystems.01291-22-s0007.docx Quality of the genomes of cefiderocol induced-resistant strains.

Table S2 msystems.01291-22-s0008.docx MICs of cefiderocol against wild-type ATCC 17978, mutants, and complemented strains.

Table S3 msystems.01291-22-s0009.docx MICs of antimicrobials against cefiderocol induced-resistant strains and recombinant strains.

Table S4 msystems.01291-22-s0010.docx Primers used in this study.

ASM does not own the copyrights to Supplemental Material that may be linked to, or accessed through, an article. The authors have granted ASM a non-exclusive, world-wide license to publish the Supplemental Material files. Please contact the corresponding author directly for reuse.
