# Supplementary material for: Mutation in the two-component regulator BaeSR mediates cefiderocol resistance and enhances virulence in Acinetobacter baumannii
Source: mSystems. 2023 Jun 22;8(4):e01291-22. doi: 10.1128/msystems.01291-22 (PMC10469669; doi:10.1128/msystems.01291-22)

**Figure S1. GO enrichment analysis of differentially expressed genes.** The top fifteen significantly enriched GO terms in ATCC 17978 BaeS^D89V^ (A) and ATCC 17978 BaeR^S104N^ (B).


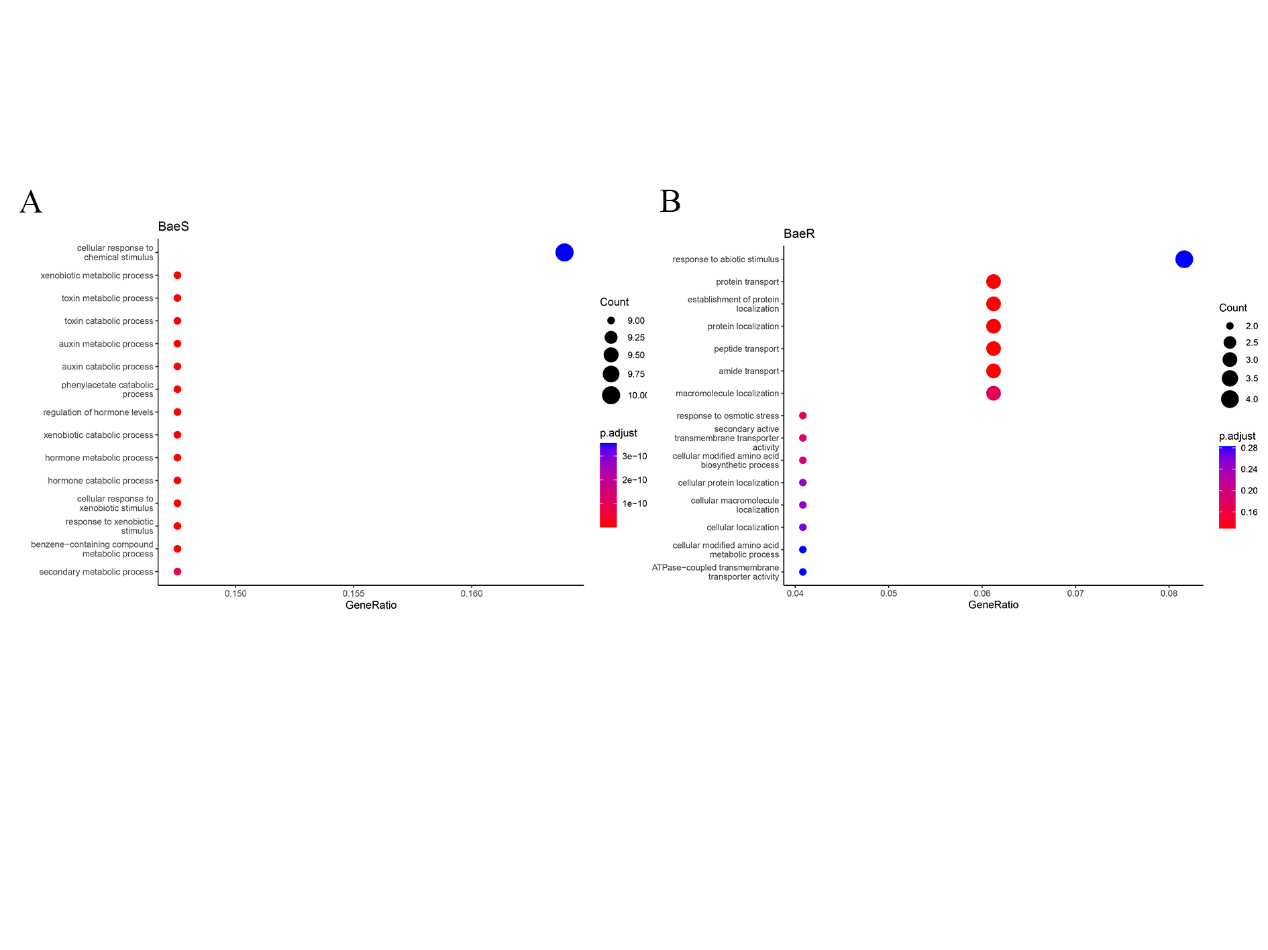

Supplement: Fig. S1 — GO enrichment analysis of differentially expressed genes. [file msystems.01291-22-s0004.docx]
