# Supplementary material for: Mutation in the two-component regulator BaeSR mediates cefiderocol resistance and enhances virulence in Acinetobacter baumannii
Source: mSystems. 2023 Jun 22;8(4):e01291-22. doi: 10.1128/msystems.01291-22 (PMC10469669; doi:10.1128/msystems.01291-22)

**Figure S2. Relative expression of AUO97_RS00560, AUO97_RS10785 and *macB* in over-expression strains.** The expression of AUO97_RS00560, AUO97_RS10785 or *macB* were significantly increased when plasmids pYMAb2 carrying target genes were introduced into ATCC 17978 compared with the wild-type ATCC 17978::pYMAb2. ***: *P* < 0.001, ****: *P* < 0.0001


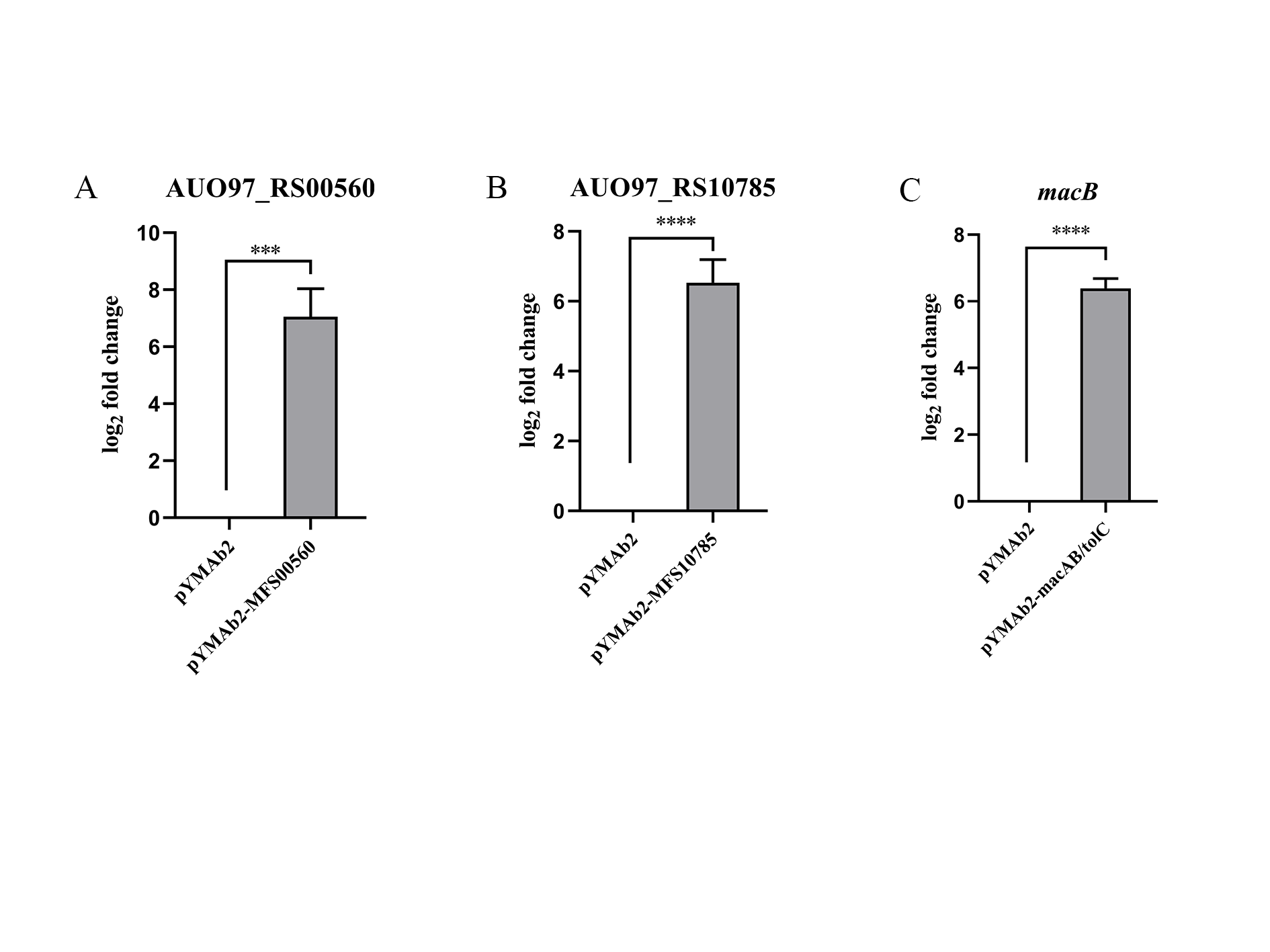

Supplement: Fig. S2 — Relative expression of AUO97_RS00560, AUO97_RS10785, and macB in over-expression strains. [file msystems.01291-22-s0005.docx]
