# Supplementary material for: Mutation in the two-component regulator BaeSR mediates cefiderocol resistance and enhances virulence in Acinetobacter baumannii
Source: mSystems. 2023 Jun 22;8(4):e01291-22. doi: 10.1128/msystems.01291-22 (PMC10469669; doi:10.1128/msystems.01291-22)

**Figure S3.** **Human bronchial epithelial (HBE) adhesion of ATCC 17978, ATCC 17978 BaeS^D89V^ and ATCC 17978 BaeR^S104N^.** (A) The counts of clones which is attached to HBE cells. (B) The HBE adhesion between BaeSR mutants and ATCC 17978 appeared no significantly different.


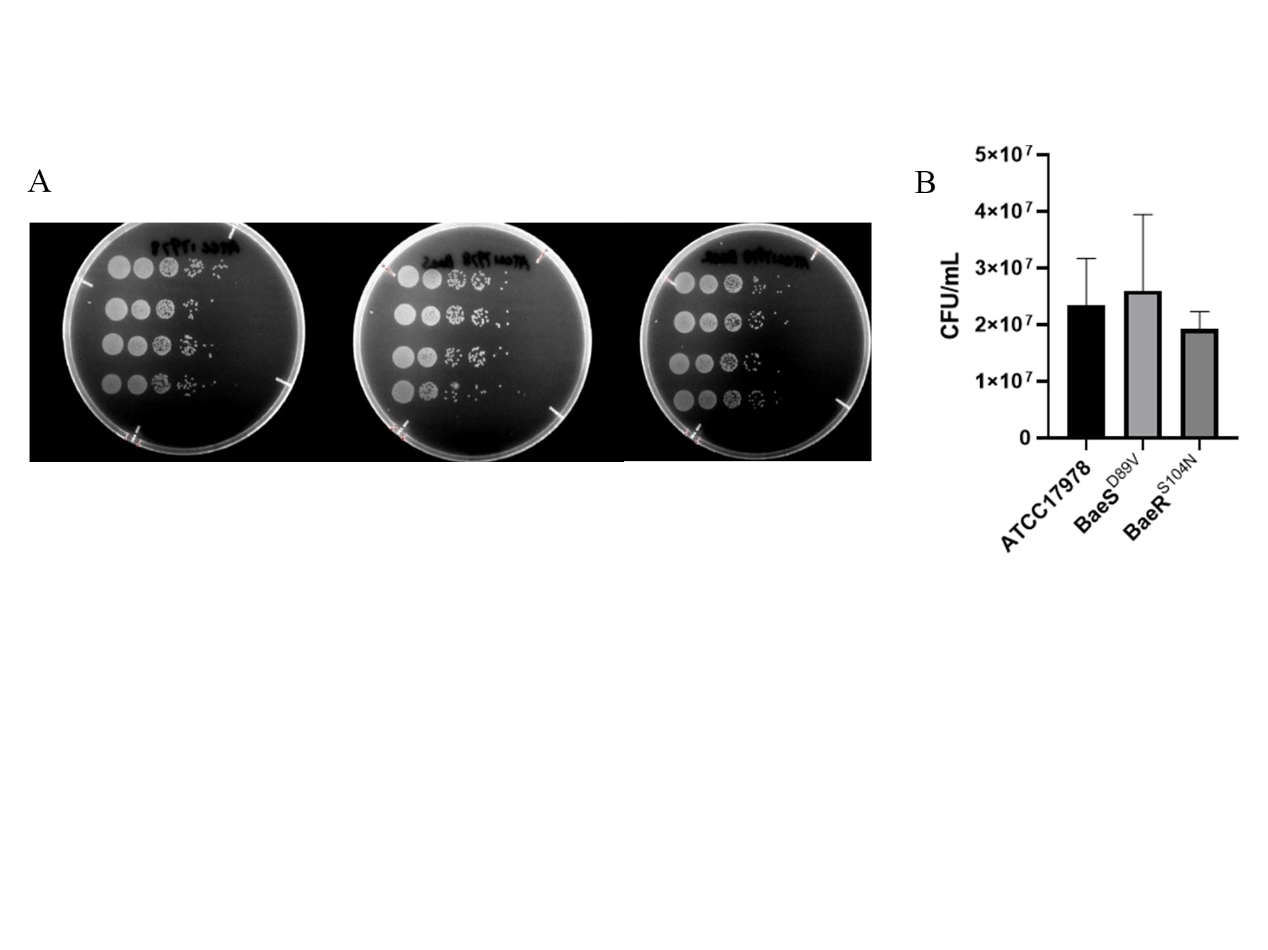

Supplement: Fig. S3 — Human bronchial epithelial (HBE) adhesion of ATCC 17978 and its BaeSR mutants. [file msystems.01291-22-s0006.docx]
