# Supplementary material for: Mutation in the two-component regulator BaeSR mediates cefiderocol resistance and enhances virulence in Acinetobacter baumannii
Source: mSystems. 2023 Jun 22;8(4):e01291-22. doi: 10.1128/msystems.01291-22 (PMC10469669; doi:10.1128/msystems.01291-22)
Supplement: Table S1 — Quality of the genomes of cefiderocol induced-resistant strains. [file msystems.01291-22-s0007.docx]

**Table S1.** Quality of the genomes of cefiderocol induced-resistant strains

| **Strains** | **Number of contigs** | **Mean read depth** | **Coverage** |
| --- | --- | --- | --- |
| XH1799 | 94 | 467.9× | 99.997% |
| XH1800 | 45 | 435.6× | 100.000% |
| XH1823 | 72 | 567.6× | 99.999% |
| XH1824 | 65 | 298.5× | 99.997% |
