# Supplementary material for: Mutation in the two-component regulator BaeSR mediates cefiderocol resistance and enhances virulence in Acinetobacter baumannii
Source: mSystems. 2023 Jun 22;8(4):e01291-22. doi: 10.1128/msystems.01291-22 (PMC10469669; doi:10.1128/msystems.01291-22)
Supplement: Table S2 — MICs of cefiderocol against wild-type ATCC 17978, mutants, and complemented strains. [file msystems.01291-22-s0008.docx]

Table S2. MICs of cefiderocol against wild-type ATCC 17978, mutants and complemented strains

| **Strains** | **FDC MIC (μg/mL)** |
| --- | --- |
| ATCC 17978 | 0.125 |
| ATCC17978 BaeS^D89V^ | 1 |
| ATCC17978 BaeR^S104N^ | 2 |
| ATCC17978ΔBaeS | 0.03 |
| ATCC17978ΔBaeR | 0.03 |
| ATCC17978ΔBaeSR | 0.03 |
| ATCC 17978:: pYMAb2 | 0.125 |
| ATCC 17978::pYMAb2-BaeS^D89V^ | 0.5 |
| ATCC 17978::pYMAb2-BaeR^S104N^ | 0.5 |
| ATCC 17978 BaeS^D89V^::pYMAb2-BaeS^WT^ | 1 |
| ATCC 17978 BaeR^S104N^::pYMAb2-BaeR^WT^ | 1 |
| ATCC 17978::pYMAb2-MFS00560 | 0.25 |
| ATCC 17978::pYMAb2-*macAB-tolC* | 0.5 |
| ATCC 17978::pYMAb2-*macAB/tolC*-MFS00560 | 0.5 |
| ATCC 17978 ΔMFS00560 | 0.125 |
| ATCC 17978Δ*macB* | 0.06 |
| ATCC 17978ΔMFS00560Δ*macB* | 0.06 |
| ATCC 17978 BaeS^D89V^ΔMFS00560 | 0.5 |
| ATCC 17978 BaeS^D89V^Δ*macB* | 0.5 |
| ATCC 17978 BaeS^D89V^ΔMFS00560Δ*macB* | 0.5 |

FDC, cefiderocol.
