# Supplementary material for: Mutation in the two-component regulator BaeSR mediates cefiderocol resistance and enhances virulence in Acinetobacter baumannii
Source: mSystems. 2023 Jun 22;8(4):e01291-22. doi: 10.1128/msystems.01291-22 (PMC10469669; doi:10.1128/msystems.01291-22)
Supplement: Table S3 — MICs of antimicrobials against cefiderocol induced-resistant strains and recombinant strains. [file msystems.01291-22-s0009.docx]

Table S3. MICs of antimicrobials against cefiderocol induced-resistant strains and recombinant strains

| **Strains** | **MIC (μg/mL)** | | | | | | | | |
| --- | --- | --- | --- | --- | --- | --- | --- | --- | --- |
|  | **Ceftazidime** | **Cefepime** | **Imipenem** | **Meropenem** | **Tigecycline** | **Polymyxin** | **Amikacin** | **Ciprofloxacin** | **Erythromycin** |
| **ATCC 17978** | 4 | 4 | 0.25 | 0.25 | 0.25 | 1 | 2 | 0. 25 | 16 |
| **XH1823** | 32 | >32 | 0.5 | 0.5 | 0.25 | 1 | 4 | 0. 25 | 16 |
| **XH1824** | >32 | >32 | 2 | 1 | 2 | 1 | >256 | 16 | >32 |
| **ATCC17978 BaeS^D89V^** | 2 | 4 | 0.25 | 0.25 | 0.25 | 1 | 2 | 0.25 | 16 |
| **ATCC17978 BaeR^S104N^** | 4 | 4 | 0.25 | 0.25 | 0.25 | 1 | 2 | 0. 25 | 16 |
| **ATCC17978ΔBaeS** | 4 | 4 | 0.25 | 0.25 | 0.125 | 1 | 2 | 0.25 | 16 |
| **ATCC17978ΔBaeR** | 4 | 4 | 0.25 | 0.25 | 0.25 | 1 | 2 | 0.25 | 16 |
| **ATCC17978ΔBaeSR** | 4 | 4 | 0.25 | 0.25 | 0.125 | 1 | 2 | 0. 25 | 16 |
