# Supplementary material for: Mutation in the two-component regulator BaeSR mediates cefiderocol resistance and enhances virulence in Acinetobacter baumannii
Source: mSystems. 2023 Jun 22;8(4):e01291-22. doi: 10.1128/msystems.01291-22 (PMC10469669; doi:10.1128/msystems.01291-22)
Supplement: Table S4 — Primers used in this study. [file msystems.01291-22-s0010.docx]

| **Table S4**. Primers used in this study | |
| --- | --- |
| **Names** | **Primer Sequences (5’→3’)** |
| BaeS-M-Veri-1-F | AACTGGTTTGCACTTAGCGG |
| BaeS-M-Veri-1-R | GTTCCCATAGCCTTGCGGTT |
| BaeS-M-Veri-2-F | CCCTGCTTGTTCGAGTTTAGG |
| BaeS-M-Veri-2-R | TTCCCGCTAAGTGCAAACC |
| BaeR-M-Veri-F | GGCGAAATTCTGTTGGCGTT |
| BaeR-M-Veri-R | ATCTTGAGGCTGCTGGGTTC |
| **Reconstruction of mutations** | |
| BaeS-F | ACCTGACCTGAGCggccgccctgcagcggatccGCCTAGTCCTGTTCCACCTGTACT |
| BaeS-R | TGCCGGGAGCAGACAagcccgtcgcatgcatctagaGACAAGCAGCCAAACTACCCA |
| Inveri-BaeS-F | GAACCCAGCAGCCTCAAGAT |
| Inveri-BaeS-R | AGTGTTCTGCACTGGACGATG |
| BaeR-F | GTGCTGACCTGACCTGAGCggccgccctgcagcggatccGATGGGCGAGCAGTTTTACG |
| BaeR-R | TGCCGGGAGCAGACAagcccgtcgcatgcatctagaGATGCCAAACACCAGTTCGTAGT |
| Inveri-BaeR-F | ACGGACGTGGCTTACTCATT |
| Inveri-BaeR-R | GTCGAGTTAGCGCATCTGGT |
| **Gene knockout** |  |
| MFS560-UP-F | GACCTGAGcggccgccctgcagcggatccCCTTATATCGGAGCTGGCGTG |
| MFS560-UP-R | TGCGCCCACATTTCCACCTgcgccaataatgGCAAGCAAACCATGCGAAG |
| MFS560-DW-F | TTGTCTGCTTCTTCGCATGGTttgcttgcCATTATTGGCGCAGGTGGA |
| MFS560-DW-R | GAGCAGACAagcccgtcgcatgcatctagaGTTTGGTTGCTTGGCCCTTT |
| Veri-MFS560-F | TCTTTTGGCACGTCCTTTGC |
| Veri-MFS560-R | ACACTACAGCCTGCAACCAT |
| Inveri-MFS560-F | TTTCGTTCAGTTCAGGGGGAC |
| Inveri-MFS560-R | AGCTGCATTTGCTGTGCCTA |
| macB-UP-F | CTGACCTGACCTGAGcggccgccctgcagcggatccGTTTGTCGAGTTCTGTGCGG |
| macB-UP-R | CAGCTTTCAGGTGGTCAACAGCAACGtgtgtcgTGTTGCTGCGTTTGTGTGTTC |
| macB-DW-F | CACCAATCAATGTTGAACACACaaacgcagcaacaCGACACACGTTGCTGTTGAC |
| macB-DW-R | TGCCGGGAGCAGACAagcccgtcgcatgcatctagaGACTCGACCACCCAAGAAA |
| Veri-macB-F | CTAGAGCTGGTCCGTAAGCC |
| Veri-macB-R | TGGTTGCTATTGTCGGCCAG |
| Inveri-macB-F | GGAGTGTGATTCCCGCAACA |
| Inveri-macB-R | ATTGGTGTGCGTATGGCTGT |
| BaeS-UP-F | GACCTGAGcggccgccctgcagcggatccTAGGTCCGTTACCAAGCGATG |
| BaeS-UP-R | GACCTGAGcggccgccctgcagcggatccTAGGTCCGTTACCAAGCGATG |
| BaeS-DW-F | AACATAATATGTTTCATGGGCCATTTTTccttttatAAGCACTCTCTCATTTTTACTC |
| BaeS-DW-R | GAGCAGACAagcccgtcgcatgcatctagaCGTACTGCACCGTGAGACAAAC |
| Veri-Del-BaeS-F | ACCAGATGCGCTAACTCGAC |
| Veri-Del-BaeS-R | TGCCATGTTCTGGCTTAGGT |
| Inveri-Del-BaeS-F | CACAGCGAGCTATTGAGGCA |
| Inveri-Del-BaeS-R | CCGAAAGGCAACACCTGAAC |
| BaeR-UP-F | GACCTGAGcggccgccctgcagcggatccGATGGGCGAGCAGTTTTACG |
| BaeR-UP-R | CAGAGTTAAATAAAAGgaaaaatggcccTTGCTTAGAAAAGTTATGCTGTGGA |
| BaeR-DW-F | TTATCCACAGCATAACTTTTctaagcaaGGGCCATTTTTCCTTTTATTTAAC |
| BaeR-DW-R | GAGCAGACAagcccgtcgcatgcatctagaGATGCCAAACACCAGTTCGTAGT |
| Veri-Del-BaeR-F | ACAGCGAGCTATTGAGGCAG |
| Veri-Del-BaeR-R | ATCGTGTCGATGATTCGCGT |
| Inveri-Del-BaeR-F | GGACGTGGCTTACTCATTGC |
| Inveri-Del-BaeR-R | CTTTGATGTTGCCGACCGAG |
| BaeSR-UP-F | GACCTGAGcggccgccctgcagcggatccGATGGGCGAGCAGTTTTACG |
| BaeSR-UP-R | AATATAGAGTAAAAATgagagagtgcttTTGCTTAGAAAAGTTATGCTGTGGA |
| BaeSR-DW-F | CATTGTGGTTATCCACAGCATAACTTTTctaagcaaAAGCACTCTCTCATTTTTACTC |
| BaeSR-DW-R | GAGCAGACAagcccgtcgcatgcatctagaCGTACTGCACCGTGAGACAAAC |
| pMO130-seq-F | CAACTACCCGGACCACAA |
| pMO130-seq-R | ACGGTGAAAACCTCTGAC |
| **Gene over-expression** |  |
| MFS00560-F | ATCTCTggaggatatccgcttgtcgacATGTCTTCAAATATTAATTTAGATGCAAAA |
| MFS00560-R | GCTAGCATGACTGGTGGAcagcaaatgggtcgcggatccTTAGGCTGCGGAATTGCG |
| MFS10785-F | GTTATCTCTggaggatatccgcttgtcgacATGAACAATGACGTTCAAGTAATATCG |
| MFS10785-R | GCATGACTGGTGGAcagcaaatgggtcgcggatccTTATGATTGTCTTTGCGGATCG |
| macAB/tolC-F | ATGACTGGTGGAcagcaaatgggtcgcggatccTTATTGCGGCAGTTCTTTTACAGG |
| macAB/tolC-R | ATGACTGGTGGAcagcaaatgggtcgcggatccTTATTGCGGCAGTTCTTTTACAGG |
| PY-BaeS-F | GACTGGTGGAcagcaaatgggtcgcggatccGGCCATTTTTCCTTTTATTTAACTC |
| PY-BaeS-R | ATTGTTATCTCTggaggatatccgcttgtcgacTTGAACGTTCTACGTGTTCCCA |
| PY-BaeR-F | ATTGTTATCTCTggaggatatccgcttgtcgacATGAAACATATTATGTTGGTTG |
| PY-BaeR-R | GACTGGTGGAcagcaaatgggtcgcggatccTTATTCTTCTGGATATTCGAAG |
| pYMAb-seq-F | TGGCGAGAAAGGAAGGGAAG |
| pYMAb-seq-R | CTGGTGGACAGCAAATGGGT |
| **qRT-PCR** |  |
| qpcr-MFS560-F | TGTTCGGTGTCGAGCAAACT |
| qpcr-MFS560-R | CCGCTACCCACCTCAATACC |
| qpcr-MFS785-F | CATTGGCGGTGCTTACAACA |
| qpcr-MFS785-R | ATACGCAGCACCCATCACAA |
| qpcr-macB-F | ATTGGTGGTGTACTTGGCGT |
| qpcr-macB-R | CTTGGCAGGTAAGAAGCCGA |
| qpcr-rpoB-F | AGTCACGCGAAGTTGAAGGT |
| qpcr-rpoB-R | AGCACGCTCAACACGAACTA |
